# Endovascular aortic aneurysm repair with reversed chimney graft technique in a patient with crossed fused renal ectopia: a technical note

**DOI:** 10.1093/jscr/rjab272

**Published:** 2021-06-22

**Authors:** Yohei Kawatani, Motoshige Yamasaki, Atsushi Oguri

**Affiliations:** Department of Cardiovascular Surgery, Takasaki Heart Hospital, Takasaki, Japan; Department of Cardiovascular Surgery, Takasaki Heart Hospital, Takasaki, Japan; Department of Cardiology, Takasaki Heart Hospital, Takasaki, Japan

## Abstract

Crossed fused renal ectopia is a very rare congenital ectopia and poses great challenges when performing abdominal aortic surgery because of the accompanying abnormal vessels and urinary tracts. A 79-year-old woman with an abdominal aortic aneurysm and L-shaped crossed fused renal ectopia was referred to our facility. One of the large ectopic renal arteries arose from the right common iliac artery. The aneurysm was treated with an endovascular aortic repair. The reversed chimney graft technique was applied to preserve the ectopic renal artery while elongating the distal landing zone on the right side. The patient experienced no complications such as renal dysfunction or recurrence of the abdominal aortic aneurysm during the 6-month follow-up period.

## INTRODUCTION

Crossed fused renal ectopia is a genitourinary anomaly in which the kidneys are fused and located on the same side of the midline. This anomaly is rare and reported to affect 1 in 7000–7500 individuals [1, 2]. Coexisting aberrations pose great challenges when performing abdominal aortic surgery [3].

## CASE REPORT

**Figure 1:**
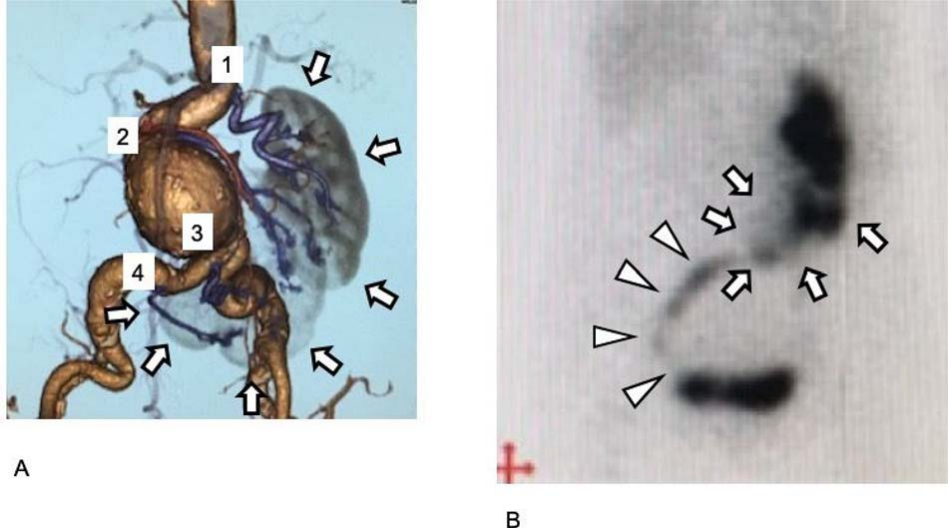
Preoperative images of the aneurysm and the kidney. (**A**) Preoperative three-dimensional enhanced CT image. Four RA are visible (purple and numbered). Cross-fused renal ectopia was diagnosed, and the right kidney had migrated to the left side. The kidney is L-shaped and indicated in gray (arrow). (**B**) Preoperative 99mTc-MAG3 renogram. The L-shaped kidney is well observed (arrow). The urinary tract arises from the left side of the kidney and progresses to the right side, and then finally runs into the bladder at the right side (arrowhead).

A 79-year-old woman with a history of surgery for peritonitis was referred to our hospital to treat an abdominal aortic aneurysm (AAA). Enhanced computed tomography (CT) revealed an AAA measuring 53 mm in diameter (Fig. 1A). The right kidney was located to the left of the midline; this condition is called L-shaped crossed fused renal ectopia (Fig. 1A and B). The patient had four renal arteries (RA): the most proximal arose from almost the normal position of the left RA; the second arose from the AAA sac; the third arose from the right common iliac artery (CIA), 7 mm distal to the edge of the AAA; and the fourth RA arose from the right CIA, 40 mm distal to the aorta (Fig. 1A). The diameters of the RAs were 6.0, 2.6, 5.6 and 3 mm in the order of the most proximal to the most distal.

**Figure 2:**
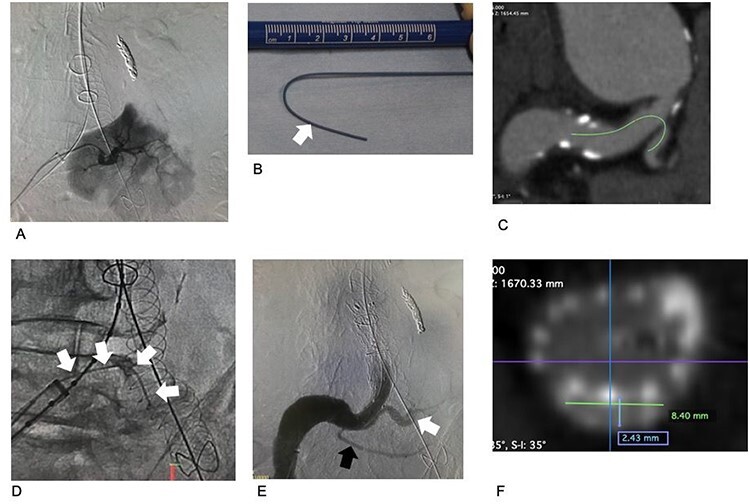
Images associated with chimney graft technique procedures. (**A**) Selective arteriogram of the third renal artery. The area perfused by the artery is large. (**B**) Before insertion, a stiff wire was shaped according to the preoperative CT image analysis. The tip of the stiff portion is indicated by a white arrow. The portion distal to the white arrow is more pliable, preventing damage to the third renal artery and the kidney. (**C**) Preoperative CT. The estimated access route for the chimney graft and the stiff wire is indicated by a green line. (**D**) The stiff wire is placed into the third renal artery. (**E**) Arteriogram after completing all procedures. The third renal artery is perfused via the covered stent (white). The fourth renal artery is preserved (black). No gutter-related type II endoleak occurred. (**F**) Postoperative CT. The cross-section of the chimney graft is 2.4 × 8.4 mm.

Surgery was performed under general anesthesia using the cut-down direct Seldinger method on the common femoral artery’s bilateral approach. First, we embolized the second RA using two 4 mm × 15 cm coils (Penumbra Coil, Penumbra, Inc., CA). A stent-graft main body (Aorfix, Lombard Medical Ltd, Oxfordshire, UK) was placed from the abdominal aorta just distal to the first RA to the left CIA. An 18-Fr dry seal sheath was placed in the right CIA. The third RA, arising from the right CIA, was selectively cannulated (Fig. 2A) using a Judkins Right-shape catheter, and a stiff wire (Super Stiff wire, Boston Scientific Corporation, Marlborough, MA) was inserted into the artery through the catheter. Before insertion, the guide wire was shaped appropriately to the curvature of its course according to the preoperative CT image (Fig. 2B–D). An iliac limb prosthesis of 16 × 89 mm (Endurant stent graft, Medtronic, Santa Rosa, CA) was deployed from the contralateral gate of the stent graft main body to just proximal to the orifice of the third RA in the right CIA.

**Figure 3:**
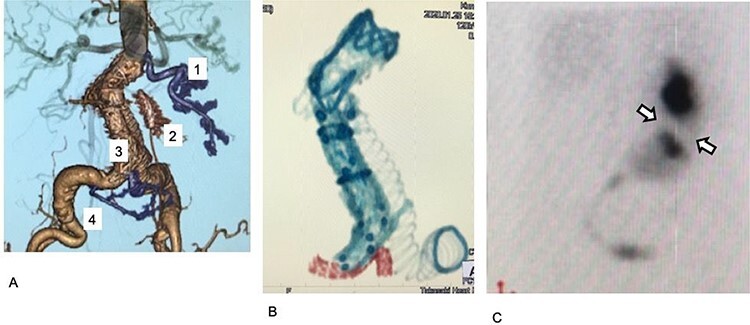
Postoperative images. (**A**) Postoperative enhanced CT. The first, third, and fourth RA are preserved (purple). The third renal artery is preserved by a chimney graft with good perfusion. (**B**) Stent grafts and the covered stent on postoperative plane CT. The covered stent is shown in red. The stent tracks well to the acute angle of the third renal artery. (**C**) Postoperative 99mTc-MAG3 renogram. Collection ceased in a small region in the kidney, which was assumed to be perfused by the second renal artery (arrow).

Subsequently, the reversed chimney graft technique was performed to preserve the perfusion of the third RA while elongating the landing zone. A covered stent (6 mm × 59 mm, Viabahn VBX covered stent, LG GORE, Delaware, USA) was inserted into the third RA using the stiff wire and was then deployed at normal pressure (6 atm). An additional iliac limb prosthesis of 16 × 89 mm (Endurant stent graft) was placed from the previously placed iliac limb prosthesis to the CIA. The third RA’s perfusion was preserved adequately, and no endoleaks were shown on the enhanced CT or the 99mTc-MAG3 renogram (Fig. 2D and E; Fig. 3A–C).

The patient recovered uneventfully, and their creatinine level did not increase. During the 6-month follow-up period, the patient progressed well without the recurrence of AAA or renal dysfunction.

## DISCUSSION

The surgical treatment of AAA in patients with crossed fused renal ectopia is made difficult by coexisting aberrations of the RA, veins and urinary tracts [3, 4]. Enhanced CT was essential for patient evaluations in this case, and the renogram was also very useful (Fig. 2A and B). RA aberrations were the most important factor in our surgery. The first and fourth RAs were preserved using the ordinary endovascular aneurysm repair (EVAR) procedures.

The second RA arose from a proximal position of the AAA, with a small diameter of 2.5 mm. The Society for Vascular Surgery recommends preserving RAs with a diameter >3–4 mm [5], and Schneider *et al*. [6] performed EVAR in a patient with crossed fused renal ectopia with embolization of a larger RA and observed only a mild decrease in renal function. Thus, in our case, the embolization of the second RA was deemed acceptable.

The third RA was large, with a diameter of 5.6 mm, and perfused a large area of the kidney (Fig. 2A). To preserve the RA and gain a sufficient distal landing zone on the right side, we performed the reversed chimney graft technique. First, an iliac limb prosthesis was placed using the conventional method from the contralateral gate to just proximal to the third RA. Second, the iliac limb prosthesis and chimney graft were placed in the right CIA.

Chimney graft, also known as snorkel or periscope, uses bare-metal or covered stents in aortic side branches parallel to and outside the main endograft [7]. This allows a total endovascular approach to an aortic disease in patients with short landing zones caused by aortic branches [8–10]. There are a few reports on the chimney graft technique’s use in EVAR in patients with crossed fused renal ectopia to protect the ectopic RA. Kfoury *et al*. [9] and Colacchio *et al*. [10] placed self-expanding endografts (Viabahn Endoprosthesis, LG GORE, Delaware, USA) into RAs that arose from the AAA sacs in their cases. Here, we opted for the reversed chimney graft technique using Viabahn VBX, a balloon-expanding endograft. We chose this endograft because the prosthesis’s stent has a stronger radial force than the self-expanding type. We assumed that this would help maintain the patency at the lesion in which the chimney graft and aortic stent graft were placed parallel in the CIA. Finally, postoperative evaluation of the chimney graft revealed an elliptical cross-section of 2 × 8 mm; thus, we assumed that the blood flow was well preserved (Fig. 3E). Postoperative enhanced CT and renogram confirmed the artery’s perfusion via the chimney graft.

In our case, the acute angle at the third RA’s origin posed a great challenge (Figs 1C and 3F). When placing Viabahn VBX, the guiding sheath should be inserted into the target vessel. We planned to use a 6-mm diameter Viabhn VBX; therefore, we should have placed a 7-Fr guiding sheath into the third RA over an acute angle at the artery’s origin, which was assumed to be very difficult. To overcome the problem, we used a stiff guide wire instead of the usual more pliable wire. Before insertion, the stiff wire was shaped according to the preoperative analysis of the access route (Fig. 3E). With the strong backup of the pre-shaped stiff wire, we inserted Viabahn VBX, without insertion of the guiding sheath, into the third RA.

In conclusion**,** EVAR with reversed chimney graft technique can be a useful treatment option for AAA in patients with crossed fused renal ectopia.
